# Implementation fidelity of a Brazilian drug use prevention program and its effect among adolescents: a mixed-methods study

**DOI:** 10.1186/s13011-022-00496-w

**Published:** 2022-11-01

**Authors:** Julia D Gusmoes, Rodrigo Garcia-Cerde, Juliana Y Valente, Ilana Pinsky, Zila M Sanchez

**Affiliations:** 1 Departamento de Medicina Preventiva, Universidade Federal de São Paulo, São Paulo, Brazil; 2 Urban Food Policy Institute, University of New York (CUNY), New York, United States of America

**Keywords:** Implementation fidelity, Prevention, Mixed-methods, Adolescent, School, Drugs

## Abstract

**Background:**

Based on the US DARE-kiR, a version of the Keepin’ it REAL program, the Drug and Violence Resistance Educational Program (PROERD) is the most widely implemented Brazilian prevention program. It originates from the translation of the DARE-kiR, a version of the Keepin’ it REAL program. Previous results suggest its inefficiency in preventing drug use among Brazilian adolescents. Since kiR fidelity can impact program outcomes, this mixed-methods study evaluates the PROERD implementation fidelity and its effects on preventing drug use among adolescents.

**Methods:**

Data from two cluster randomized controlled trials (cRCTs) with 4,030 students from 30 public schools in São Paulo (1,727 fifth graders and 2,303 seventh graders), assessed at two-time points, were analyzed quantitatively. After implementing each lesson during the cRCT, 19 PROERD instructors answered fidelity forms. The effect of PROERD fidelity on alcohol, cigarettes, marijuana, inhalant, and cocaine use (the last two only among seventh graders) in the six months prior to follow-up assessment was analyzed by logistic regressions for fifth grade and mixed effect models for seventh graders. For qualitative analysis, semi-structured interviews were conducted with PROERD instructors and investigated by thematic analysis.

**Results:**

Quantitative analysis showed that PROERD implementation fidelity had no impact on drug use among fifth and seventh graders. Conversely, the qualitative analysis revealed important aspects that may influence implementation fidelity and consequently program effectiveness, such as adaptations made by instructors, school infrastructure, among others, besides program application.

**Conclusion:**

PROERD requires cultural adaptation to improve its implementation in Brazilian public schools.

**Supplementary Information:**

The online version contains supplementary material available at 10.1186/s13011-022-00496-w.

## Introduction

Adolescence is the typical period during which initial alcohol and drug use occurs [1], impacting mental health [2] and facilitating the development of drug-related issues [3], as well as drug dependence in adulthood [4]. Hence, alcohol use in early adolescence is an important public health concern [5], contributing to the global disease burden [6].

Among Brazilian adolescents between 13 and 15 years of age, 55.5% reported alcohol consumption and 9.0% had used illicit drugs at least once in their lives, whereas 22% reported episodes of drunkenness [7]. Despite being a major public health concern, few evidence-based programs for drug use prevention have been implemented and proven efficacious in Brazilian schools [8]. The Drug and Violence Resistance Educational Program (*Programa Educacional de Resistência às Drogas e à Violência* – PROERD) is the most widely implemented school-based prevention curricula, reaching approximately 40% of schools [8]. A recent study showed that PROERD failed to achieve better preventive drug outcomes in the intervention group compared to the control group for both 5th and 7th grades [9]. Moreover, the program appears to negatively impact secondary outcomes, as the seventh grade curriculum seems to increase the intention to use cigarettes in the future and chances of accepting marijuana, whereas the fifth grade curriculum slightly reduces decision-making skills [10].

Its current curriculum is a Brazilian Portuguese translation of the US DARE-Keepin’it REAL (DARE-kiR) program [11], renamed as “PROERD-Caindo na Real,” and implemented by military police officers in all Brazilian states. Of wide implementation in US schools, DARE-kiR is also enforced by the military police. However, no studies have reported its impact on drug use prevention [12]. The only published paper reporting DARE-kiR findings is a quasi-experimental matched group study that looked at its impact on secondary outcomes [11]. Prior to adaptation by DARE, kiR was originally designed and developed to be implemented in 7th grade classrooms by their teachers. The original program was tested with 7th graders in Arizona-USA [13], and its culturally adapted versions were also evaluated by multiple RCTs in the US and in other countries [14–17], showing consistently positive results. As the kiR program for fifth graders proved to be ineffective, its developers recommended continued intervention only for 7th grades [18, 19]. Randomized control trial (RCT) evaluations of the kiR seventh grade curricula have shown consistently favorable results on drug use prevention in the US [13], Guatemala [14], Mexico [15], and Spain [17]. RCT evaluations of the kiR seventh grade curriculum have reported positive outcomes for discontinuing alcohol use [20] and intoxication episodes [17], as well as preventing cigarette [16, 21], marijuana [14, 16, 22] and other illicit drugs [15, 22] use. Conversely, kiR fifth grade curricula remain poorly investigated, and the only RCT reported a significantly increase in the prevalence of substance use over the 3-year period [19]. Despite the scarcity of reports on DARE-kiR implementation, some studies measured and reported findings on kiR fidelity. Marsiglia et al [23, 24] reported findings on fidelity in the context of cultural adaptation of the program to the Mexican population, as well as Cutrin to the Spanish population [17]. The only study that we are aware of that has investigated the impact of kiR implementation fidelity on programs outcomes found that the program’s impact on positive outcomes can increase based on delivery quality [25]. PROERD and DARE-kiR are a translated version of the same program, share the same theoretical model of their original version (kiR program). The main difference between the programs is that kiR is implemented by teachers, and DARE-kiR and PROERD are implemented by police officers.

Considering these unexpected results from the Brazilian version of the DARE-kiR program, we must investigate which factors might be affecting the outcomes. One factor that reportedly impacts a prevention program’s effectiveness is the excellence (or lack thereof) of its implementation [26], which can be determined by implementation fidelity, that is, the degree to which an intervention and its core components are delivered as intended by the program developers [27, 28]. A crucial aspect of implementation, that provides important information for measuring fidelity, especially when the program is not implemented by researchers, is the dosage, that is, the amount of program delivered [29]. In a review, Hill and Erickson [30] found that implementation fidelity plays an important role in program outcomes, as programs delivered with high or moderate levels of fidelity show more than double the potential to achieve positive results, contradicting the null outcome seen in our study. Despite the scarcity of reports on DARE-kiR implementation, a study investigating kiR implementation fidelity found that its impact can increase based on the quality of delivery [25].

According to Pettigrew et al. [31], teachers may adapt the program to their teaching characteristics. Such adaptation of evidence-based programs, that is, modifying the design or delivery of an intervention to address cultural and contextual specificities, can impact their results [32]. However, the relation between fidelity and adaptation remains controversial, with some researchers arguing that practices implemented with high fidelity result in better outcomes [33], while others highlight the importance of balancing between fidelity and flexibility for a successful interventions [34]. These findings point to the importance of identifying aspects that may require adaptation, as previously done among populations in Guatemala [35] and Mexico [23], as well as in rural communities in Pennsylvania and Ohio [25].

Given the relevance of implementation fidelity to better program outcomes, we hypothesized that PROERD delivered with high fidelity would have a better effect among students. Hence, this study sought to evaluate, by quantitative and qualitative methods, the PROERD implementation fidelity and its effect on drug use prevention.

## Methods

Of a mixed-methods design, the study obtained quantitative data from two cluster randomized controlled trials (cRCTs) and collected qualitative data using (1) questionnaires applied to cRCTs participants, (2) fidelity forms answered by instructors (police officers) after each cRCT lesson, and (3) semi-structured interviews conducted with the instructors who delivered lessons. The interviews and qualitative analysis allowed us to answer research questions and clarify cRCT findings and quantitative results [36].

### Intervention

The school-based “PROERD-Caindo na Real” program consists of 10 weekly classes (50 min each) delivered by trained police officers in the classroom environment, using student and teacher manuals. The police officer responsible for teaching the class uses the teacher’s manual, which provides information on procedures, objectives, materials needed, and tips for each lesson, including 1–3 activities. All participating officers underwent 80 h of training offered by the Military Police under the guidance of DARE America. Fifth and seventh graders were taught by the same instructor. All curricula were developed based on theories of narrative engagement [37], the principle of cultural grounding [38], social and emotional learning [39], and normative beliefs on drug use [40]. In Brazil, the program is implemented by the Military Police. Despite the lack of information about the program’s cultural adaptation process, a comparative reading of the DARE-kiR and PROERD manuals suggests that the latter is simply a translation into Brazilian Portuguese of the DARE material, lacking cultural and socio-environmental elements specific to the Brazilian context. Thus, its effectiveness needs to be assessed considering this factor.

### Quantitative methods

#### cRCT study design

Two parallel two-arm cRCTs were conducted with 4,030 fifth and seventh graders from 30 public schools in São Paulo in 2019 to evaluate the PROERD curricula for drug use prevention. Of these, 1,727 were fifth graders enrolled in 72 classes at 28 schools, and 2,303 were seventh graders enrolled in 90 classes at 30 schools. Both intervention groups attended 10 classes taught by 19 trained police officers; the control group received no intervention. State schools in the municipality of São Paulo that offered 5th and 7th grade and had not received PROERD in the last three years were included in the randomization. The first 30 schools listed were considered the study sample and the following 29 schools were included as backup in case of refusal. In the schools that agreed to participate in the study, all 5th and 7th graders participated in the cRCT. Sample size calculation, school selection, and the randomization process were performed according to Valente & Sanchez [10].

#### First data source: questionnaires applied to cRCT participants

Data were collected at two-time points. Baseline assessment was conducted prior to program implementation between February and March 2019. As the Brazilian academic year usually runs from February to December, follow-up data were collected 9 months after baseline in November and December 2019. Control and intervention data were collected simultaneously. An anonymous, self-administered audio-guided questionnaire was applied to students using smartphones by researchers in the classroom without a teacher. This instrument has been employed in previous studies to evaluate school-based drug prevention programs in Brazil [41, 42]. It was designed based on the European Drug Addiction Prevention Trial (EU-DAP) questionnaire [43], translated and adapted into Brazilian Portuguese. We added a few questions from the World Health Organization (WHO) questionnaire, used in the VI Brazilian Survey on Drug Use among Students [44], and the National Survey of School Health (PENSE) questionnaire, from the Ministry of Health [45].

The outcome analyzed was the prevalence of drug use among fifth and seventh graders in the past 6 months before the follow-up assessment (yes or no), including alcohol, tobacco, marijuana, binge drinking, inhalants, and cocaine (the last two assessed only among seventh graders). During follow-up, the adolescents answered questions such as “Have you drunk alcoholic beverages in the past six months?” Binge drinking was considered as the consumption of five or more alcoholic beverages on a single occasion.

Control variables consisted of sex (male/female), age, and socioeconomic status (SES). SES was assessed by the Brazilian Association of Research Companies (ABEP) scale, which considers the schooling level of the head of the household and the goods and services used. ABEP score ranges from 1 to 100 points, graded from A (highest) to D/E (lowest) according to established cutoff points: A (45–100 points), B (29-44 points), C (17-28 points), and D/E (0–16) [46].

In each assessment, students provided a code generated from letters and numbers of their personal information, which allowed to match pre- and post-tests, ensuring anonymity and confidentiality, as used previously in drug prevention program evaluations [47]. Since some students may overreport their drug use, we included questions related to fictional drugs called “holoten” and “carpinol.” Questionnaires positive for lifetime use of these drugs were excluded from the analysis (14 and 12 questionnaires at baseline and 11 and 8 questionnaires at follow-up for fifth and seventh graders, respectively).

#### Second data source: fidelity forms answered by instructors

Data on implementation fidelity were collected using self-administered online questionnaires completed by the instructors after each lesson, reporting whether the scheduled activities were delivered (yes or no) and whether any activities were altered by the instructors (yes or no). The fidelity forms listed all activities planned for each program lesson, based on the teacher’s manual. The first author (J.D.G.) trained the police officers on how to fill the form. Each item had data on the percentage of activity completeness (the numerator was the number of activities delivered, and the denominator was the total number of activities planned), and percentage of alterations (the numerator was the activities instructors reported changing, and the denominator was the number of activities planned). A fidelity variable for each class was calculated as follows: fidelity = % completeness× (1-% alteration). Classes were then divided into two groups according to the level of fidelity: those that received ≥ 80% of the proposed PROERD activities were considered to have completed the program, whereas those that received ˂80% of the activities were considered to have incomplete implementation.

Since we evaluated 35 fifth grades and 47 seventh grades, and awaited 10 fidelity forms from each class (1 for each of the 10 lessons delivered), we expected a total of 350 and 470 forms for fifth and seventh grade classes, respectively. However, some instructors failed to return the forms, resulting in 47 (13%) and 83 (17,66%) fifth and seventh grade forms not delivered and considered missing. Online completion allowed researchers to mark all responses as “mandatory,” thus avoiding missing answers. Completeness and alteration calculations were proportional to the information available without missing data.

### Qualitative methods

After the intervention, data were collected by semi-structured interviews conducted with all 19 PROERD instructors involved in the cRCT (19 instructors delivered the program in 30 intervention schools).

#### Third data source: semi-structured interviews with instructors

Qualitative data were collected by semi-structured in-depth interviews [48], with a set of previously defined questions, to which the interviewer was free to add new questions if necessary. To reduce interviewer interference and facilitate data organization, comparison, and analysis, all interviewees were asked the same basic set of questions [49].

Interviews lasted around 45 min and touched on the following topics: (i) how and why the participants became a PROERD instructor; (ii) how they perceive the effects (if any) of the program; (iii) their relationship with the school counselor and how it could affect the program’s impact; (iv) whether they considered the program training sufficient and how it influenced their work in PROERD; (vi) their opinions on the material and its content; (vii) what planned activities were well and poorly received; (viii) differences in applying the curricula for fifth and seventh graders; (ix) how PROERD demands affected other officer duties and vice versa; (x) what they would change in the program; (xi) what could be done to improve PROERD implementation. All interviews were recorded with prior consent from the interviewees. The resulting data were anonymized, transcribed verbatim, and identified by an alphanumeric code combining the letter (P), for police officer, and a random number assigned according to the order of the interviews (01, 02, 03…).

### Data analysis

#### Quantitative analysis

Sociodemographic characteristics and drug use data underwent descriptive analysis, with categorical variables expressed as numbers and percentages, and numerical variables expressed as means and standard deviations. We then performed inferential analysis to assess the impact of PROERD implementation fidelity on reducing adolescent drug use. For seventh graders, the impact of PROERD implementation fidelity on alcohol, cigarette, marijuana, inhalants, and cocaine use and binge drinking in the past 6 months was examined by a mixed-effects linear model. This model considers variability within individuals (from baseline to follow-up) and between individuals (children nested in schools), highlighting the relations between the observed response and explanatory covariates [50, 51]. Given the extremely low prevalence of drug use among fifth graders, this impact was assessed using logistic regressions. All analyses considered 0 as control group, 1 as low fidelity, and 2 as high fidelity. Analysis was performed using STATA software version 17.0 and adjusted for sex, age, and SES, considering non-independence of the sample (children nested in school).

Data obtained from the fidelity forms on the completeness and alterations of PROERD lessons were underwent descriptive analysis, summarized as numbers and percentages.

#### Qualitative analysis

Data underwent thematic analysis [52] using axial coding, in which a priori analytical categories were generated based on interview guide topics, subsequently linked to other subcategories along the lines of their properties and dimensions [53]. Of the 25 codes identified, we chose to analyze 8 codes based on their possible influence on PROERD effectiveness (see Figure S1), organized into 3 topics: (1) adaptations, (2) accumulation of functions, and (3) school infrastructure. Qualitative analysis was performed using ATLAS.ti © version 7.5.4.

After initial coding performed by the first author (J.D.G.), a PhD candidate in public health with training and experience in qualitative analysis, data underwent interpretive triangulation by the second author (R.G.C.), an anthropologist and PhD candidate in collective health, who analyzed the data in parallel. Disagreements were resolved by discussions, and a second review of the interview transcripts. Topics reported and discussed in this manuscript resulted from a consensus among researchers.

## Results

### Quantitative analysis

**Fig. 1:**
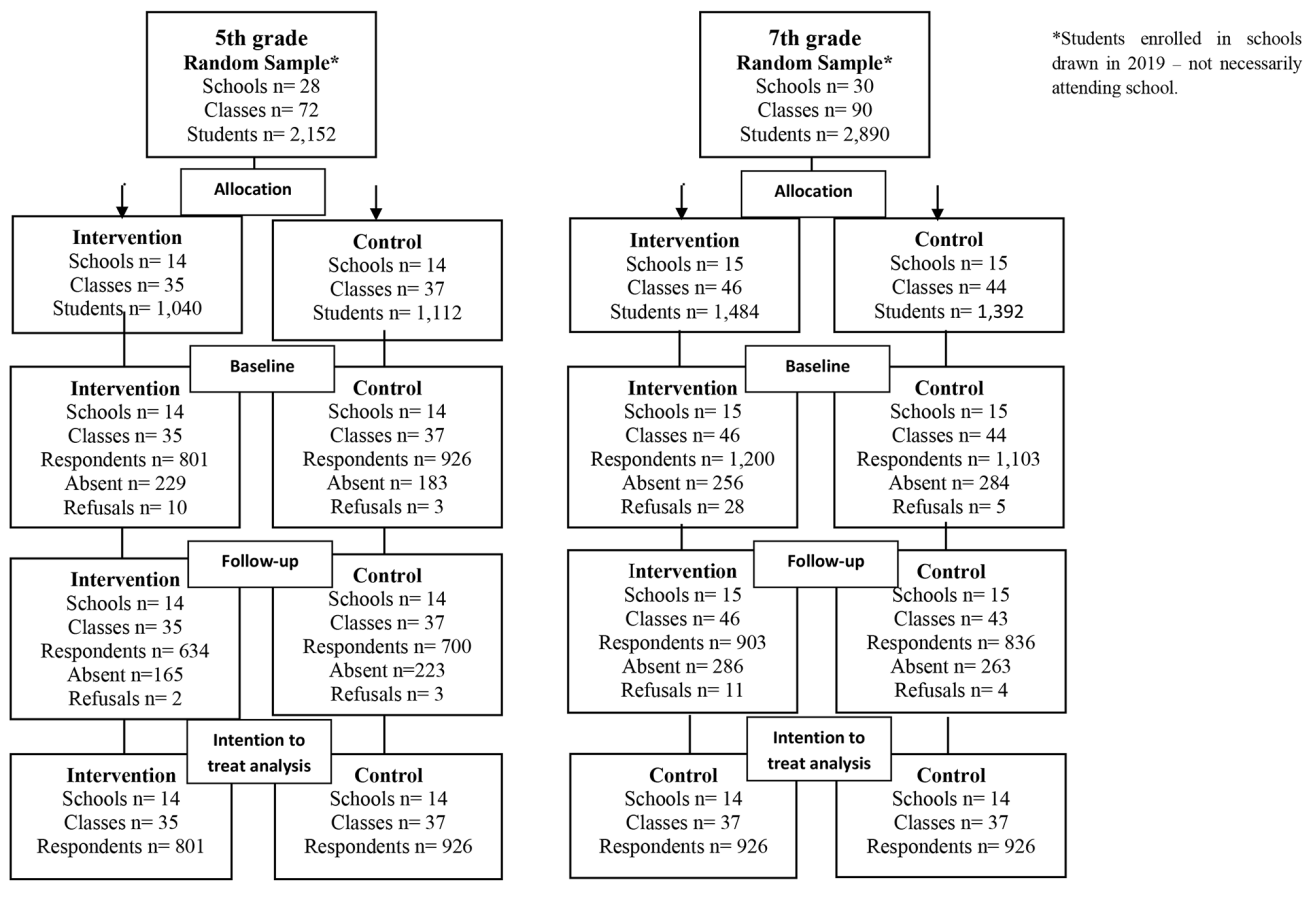
Flowchart of the randomized controlled trial assessing the effect of the PROERD drug use prevention program among 5th and 7th graders

Among fifth graders, 1,727 students answered the baseline questionnaire and 1,334 the follow-up questionnaire (77.24%). Among seventh graders, 2,303 students answered the baseline questionnaire and 1,739 the follow-up questionnaire (75.51%) (Fig. 1).

Table 1 summarizes the characteristics of the fifth and seventh graders who participated in the cRCT baseline assessment. At baseline, the intervention and control groups were homogenous in terms of sex, age, and SES (see Table S1 and Table S2 in the Supplementary File). The attrition analysis found no significant difference between groups and between sex. As expected, however, students who missed the 9-month follow-up showed a significantly higher prevalence of substance use at baseline, especially among 7th graders [42]. Table 2 presents the descriptive results of drug use at follow-up according to group and level of implementation fidelity. Alcohol was the most commonly used drug in both grades and groups.

**Table 1:** Distribution of 5th and 7th graders according to sociodemographic data, drug use (alcohol, binge drinking, tobacco and marijuana) and allocation group in the cluster randomized controlled trial of the PROERD program, according to baseline. Brazil, 2019 (N = 4,030; 1,727 5th and 2,303 7th graders)

	Total		Intervention		Control	
	**N**	**%**	**N**	**%**	**N**	**%**
**5th grade students (N = 1,727)**			**(N = 801)**		**(N = 926)**	
**Gender**						
Male	882	51.07	432	53.93	450	48.60
Female	845	48.93	369	46.07	476	51.40
**Age (mean** **±** **SD)**	10.12 ± 0.65		10.10 ± 0.68		10.14 ± 0.61	
**SES** ^**a**^						
A	117	9.00	49	7.94	68	9.96
B	447	34.38	224	36.30	223	32.65
C	646	49.69	309	50.08	337	49.34
D-E	90	6.92	35	5.67	55	8.05
**Alcohol**						
Past year Use	161	9.36	81	10.18	80	8.65
**Binge drinking**						
Past year Use	20	1.16	9	1.13	11	1.19
**Tobacco**						
Past year Use	12	0.70	6	0.75	6	0.65
**Marijuana**						
Past year Use	4	0.23	1	0.13	3	0.33
**7th grade students (N = 2,303)**			**(N = 1,200)**		**(N = 1,103)**	
**Gender**						
Male	1,187	51.54	621	51.75	566	51.31
Female	1,116	48.46	579	48.25	537	48.69
**Age (mean** **±** **SD)**	12.28 ± 0.72		12.28 ± 0.74		12.27 ± 0.71	
**SES** ^**a**^						
A	130	5.71	74	6.25	56	5.12
B	773	33.93	416	35.14	357	32.63
C	1,222	53.64	629	53.13	593	54.20
D-E	153	6.72	65	5.49	88	8.04
**Alcohol**						
Past year Use	442	19.26	234	19.57	208	18.93
**Binge drinking**						
Past year Use	132	5.77	71	5.95	61	5.58
**Tobacco**						
Past year Use	36	1.57	20	1.67	16	1.46
**Marijuana**						
Past year Use	41	1.79	22	1.84	19	1.73
**Inhalants**						
Past year Use	57	2.49	31	2.60	26	2.37
**Cocaine**						
Past year Use	2	0.09	0	-	2	0.18

^a^ SES: Socioeconomic status according to ABEP Scale – A (45 to 100 points), B (29 to 44 points), C (17 to 28 points) and D/E (0–16 points), where A is the highest and E the lowest

**Table 2:** Descriptive fidelity and drug use^c^ in the follow-up assessment

	Control		Intervention			
			**Low fidelity** ^**a**^		**High fidelity** ^**b**^	
	**N**	**%**	**N**	**%**	**N**	**%**
**5th grade students (N = 926)**			**(N = 513)**		**(N = 288)**	
Alcohol	55	7.88	35	8.60	12	5.33
Binge drinking	4	0.57	0	0	2	0.88
Tobacco	8	1.15	2	0.49	0	0
Marijuana	1	0.14	1	0.25	0	0
**7th grade students (N = 1.103)**			**(N = 498)**		**(N = 702)**	
Alcohol	216	25.96	96	24.74	150	29.24
Binge drinking	58	6.98	26	6.70	56	10.92
Tobacco	22	2.64	13	3.35	15	2.91
Marijuana	17	2.04	8	2.06	10	1.94
Inhalants	29	3.49	8	2.07	20	3.90
Cocaine	3	0.36	1	0.26	0	0

^a^ High fidelity describes those who attended at least 80% of the activities proposed by PROERD curricula

^b^ Low fidelity describes those who attended less than 80% of the activities proposed by PROERD curricula

^c^ Drugs used in the 6 months prior to follow-up assessment

As for program implementation fidelity, 37.1% of fifth grade classes received the program with high fidelity, with lesson seven (effective communication) being the most incomplete (20% completeness) and lesson three (making choices) the most altered (17.9% alteration). Similarly, 61.7% of seventh grade classes received the program with high fidelity, with lesson ten (eco map) being the most incomplete (67.5% completeness) and lesson four (assertive refusal) the most altered (21.6% alteration) **(**Table 3**)**. We observed no effect of implementation fidelity on reducing drug use for either grade (see Tables 4 and 5).

**Table 3:** Results on the completeness and alterations made to the PROERD program based on PROERD fidelity forms

5th grade (N = 35 classrooms)	Completeness		Alterations		7th grade (N = 47 classrooms)	Completeness		Alterations	
	**N**	**%**	**N**	**%**		**N**	**%**	**N**	**%**
Lesson 1 (N = 34)	30	88.23	5	7.35	Lesson 1 (N = 43)	42	97.67	2	2.33
Lesson 2 (N = 29)	13	44.83	7	8.05	Lesson 2 (N = 43)	41	95.35	5	11.63
Lesson 3 (N = 28)	27	96.43	5	17.86	Lesson 3 (N = 37)	5	86.49	2	2.70
Lesson 4 (N = 34)	34	100	6	8.82	Lesson 4 (N = 37)	37	100	8	21.62
Lesson 5 (N = 27)	24	88.89	6	7.41	Lesson 5 (N = 36)	36	100	3	8.33
Lesson 6 (N = 30)	23	76.67	14	15.55	Lesson 6 (N = 36)	34	94.44	4	11.11
Lesson 7 (N = 30)	6	20	4	6.67	Lesson 7 (N = 38)	34	89.47	1	2.63
Lesson 8 (N = 28)	22	78.57	8	4.28	Lesson 8 (N = 37)	32	86.49	5	6.76
Lesson 9 (N = 30)	26	86.67	2	3.33	Lesson 9 (N = 40)	34	85	7	8.75
Lesson 10 (N = 33)	29	87.88	2	3.03	Lesson 10 (N = 40)	27	67.50	10	8.33

**Table 4:** Effect of PROERD fidelity over the past 6-month drug use among 5th graders* (N = 1,727)

	Implementation fidelity					
	**Low fidelity**			**High fidelity**		
	**OR**	**95%CI**	**p-value**	**OR**	**95%CI**	**p-value**
Alcohol	1.05	[0.651;1.704]	0.830	0.70	[0.359; 1.365]	0.295
Binge drinking	1	-	-	1.82	[0.309; 10.687]	0.509
Tobacco	0.46	[0.096; 2.181]	0.327	1	-	-
Marijuana	2.19	[0.120; 40.046]	0.596	1	-	-

*All analyses were adjusted by sex, age and SES.

**Table 5:** Effect of PROERD fidelity over the past 6-month drug use among 7th graders* (N = 2.303)

	Implementation fidelity					
	**Low fidelity**			**High fidelity**		
	**OR**	**95%CI**	**p-value**	**OR**	**95%CI**	**p-value**
Alcohol	0.77	[0.470; 1.253]	0.292	1.05	[0.674; 1.623]	0.841
Binge drinking	0.77	[0.357; 1.658]	0.504	1.28	[0.683; 2.404]	0.441
Tobacco	1.19	[0.447; 3.161]	0.729	1.52	[0.693; 3.353]	0.294
Marijuana	NE	-	-	NE	-	-
Inhalants	1.59	[0.601; 4.204]	0.350	0.72	[0.273; 1.904]	0.510
Cocaine	NE	-	-	NE	-	-

N.e. not estimate

*All analyses were adjusted by sex, age and SES.

### Qualitative analysis

Table S3 (Supplementary File) summarizes the interviewees’ characteristics, and Annex S1 (Supplementary File) presents the semi-structured interview script applied to police officers.

Findings were classified into three dimensions—adaptations, accumulation of functions, and school infrastructure—, corresponding to the eight codes. Table 6 summarizes the respective golden quotes.

**Table 6:** Main results from the qualitative analysis regarding the PROERD instructors participating in the study (N = 19)

Dimension	Subtopic	Description	Golden quotes
Adaptation	Adaptation according to the student literacy	Changes made by the instructors because students would not understand the purpose of the activity or lacked proper writing-reading skills.	*“The instructor has to be aware of such difficulties and adapt the activity so that group can understand. For example, they* [the students] *have to write an essay, but one student does not know how to write. That student is at a disadvantaged, right? How will they compete—I have a medal* [to give them]*—if they do not know how to write? How will they receive the medal? So, we have to evaluate them differently, as they also have the right to receive* [the medal], *to show what they learned. You can ask them “what have you learned?”, record the answer, and they will narrate, explain, and speak, and you will evaluate them along with the others* [answers]. *Hence, there has to be a different form of assessment, and you have to be ready for it.” (P10)*
	Addition of content and/or activities	Addition of content and/or activities that go beyond the suggested topic in the material.	*“We start the subject. When I get to the topic of cigarettes, I also discuss marijuana, along with cocaine and other drugs, because none have been mentioned* [in the curricula]*!” (P18)*
	Changes in the curricula	Changes made to the PROERD curricula according to the instructors’ opinions of what works or not, excluding all other reasons.	*“There are situations in the PROERD classes, during the activity, that I make changes! Changes that I think need to be done! Because if I do make changes, I believe it will not work.” (P4)*
	Exclusion of content and/or activities	Exclusion of content and/or activities because the instructor lacks the required resources.	*“I take this opportunity to mention that the issue with video demonstration is that many schools lack the material conditions to show videos, you see? Some schools lack projectors. Some schools have an unusable television in the classroom and lack a sound system, so this is a considerable difficulty.” (P10)*
Accumulation of functions	Rarely assigned other activities	Instructors supported by superiors and are rarely assigned other police activities, thus not impacting program application.	*“Our police battalion supports us. Fortunately, they assign us to other operations rather sporadically, you know? Thus, it does not influence our work considerably. We can develop the PROERD program smoothly, as individuals working with PROERD are rarely assigned other activities! For us here, it is super smooth. However, we know other places where police instructors work around the school, have to apply PROERD, and perform police operations… it doesn’t work very well. Here, for us, it is super peaceful.” (P7)*
	Assigned other activities because they take part in PROERD	Instructors who believe they are assigned more police duties because they take part in PROERD.	*“Police routine harms PROERD because the staff, the administration, and our hierarchical superiors often assume that PROERD officers, because we work during administrative hours teaching at schools, do nothing. Hence, they think our work* [with PROERD] *is very easy. They end up committing us to extra shifts—“oh, you don’t do anything anyway, so, have an extra shift”—assigning us on weekends, at different times, because they think we don’t do anything during our normal working hours, you know? And this ends up hampering* [the work with PROERD].*” (P8)*
	Assigned other activities during class hours	Instructors assigned police duties during class hours.	*“Even during class hours, they* [the superiors] *do not care. That’s the problem. They usually put us on the school round, and the school round is at the same time as our* [classes], *only we… they follow their schedule, we don’t.” (P1)*
School infrastructure	School reality	Instructors face difficulties to implement the program at public schools that lack material and media resources.	*“But the difficulty we face concerns media resources; if you need some paper, that is a resource… Yeah, supply material. There are no conditions, the school has no material conditions.” (P2)* *“What stands out is the school structure, the lack of resources and space to show* [the multimedia part of the program], *apply it, especially for seventh graders, because it* [the seventh grade curricula] *needs video, a specific room, and so many public schools lack that. It is a matter of the material and the infrastructure of the State itself.” (P2)*

Adaptations: Of the 19 interviewed instructors, 15 reported adapting the program, usually spontaneously and based on individual perception of student’s needs (without any systematics), such as reading and writing difficulties. Instructors also chose to adapt the program when lessons were thought to flow better if applied differently from the established program. We identified four types of adaptations to PROERD curricula: alterations according to student literacy, addition of content and/or activities, changes in curricula, and exclusion of content and/or activities.

Accumulation of functions: Accumulation of two functions strongly influenced the instructor’s work. Police officers responsible for implementing PROERD do not necessarily leave their regular policing duties, which hinders the planned 10-week application, as some officers may need to report for duty during class hours or because their shifts interfere with class preparation, thus impacting the intended program delivery. Only four instructors reported being rarely assigned to other activities besides the program, which facilitated their work as PROERD instructors. Of the remaining interviewees, 11 stated being assigned to other activities or experiencing a lack of support from their superiors, as they eventually missed classes due to other duties.

School infrastructure: According to the instructors, the reality of public schools in São Paulo makes it impossible to show videos included in the program’s material, resulting in adaptations to the curriculum.

## Discussion

This study analyzed the impact of PROERD implementation fidelity based on quantitative and qualitative methods. According to quantitative results, the level of implementation fidelity had no influence on the program’s ability to reduce adolescent drug use. Conversely, the qualitative analysis revealed important implementation aspects that must be considered when examining the program outcomes, such as adaptations made by the instructors, accumulation of functions (police officers who are also PROERD instructors), and school infrastructure.

Contrary to previous literature, which suggested that implementation fidelity plays an important role in program outcomes [25, 30], the present study found no influence of implementation fidelity on PROERD results. Previous studies have addressed the importance of examining the program design and contextual factors to better understand null results [54, 55]. Our null findings may be explained by the lack of cultural adaptation of the program to the Brazilian context, which is corroborated by the interviews. Moreover, compared with students in 35 other member countries of the Organization for Economic Cooperation and Development, Brazilian students’ reading performance is below average (OECD, 2019; PISA, 2016), requiring a series of adaptations to the program curricula to ensure global understanding of the proposed lessons. As PROERD appears to be simply a translation of the DARE-kiR into Brazilian Portuguese, these findings highlight the importance of a reexamination by the Military Police of São Paulo, focusing on appropriate and structured cultural adaptation [56]. In the absence of a well-designed, evidence-based cultural adaptation, instructors adapt program curricula according to their judgment, making it difficult to target core elements and consequently achieve the expected results [33].

The higher rates of low implementation fidelity suggest that instructors, when implementing evidence-based programs in schools, often encounter unpredictable situations that lead to changes in activities, such as lack of school infrastructure, affecting the level of implementation fidelity [31, 33]. Differences in implementation fidelity between fifth and seventh grades may stem from the fact that, in the state of São Paulo, PROERD is delivered mainly to fifth graders, which could lead to a greater adaptation, that is, the greater the knowledge about the curricula, the more instructors feel comfortable to adapt according to their previous experience [57]. The challenge for researchers and developers of prevention programs is therefore to define core elements that are mandatory to achieve the expected outcomes [23, 56], while allowing for some flexibility so the program can adapt its theoretical model to the local context and culture [34, 56]. As the fidelity forms used quantified only the lessons taught and ignored teaching quality [58], the quantitative analysis failed to assess important aspects of implementation. Hence, a mixed-methods approach allows one to evaluate details that would be lost when only one method is used [59].

Qualitative data revealed that instructors adapted the program curricula according to aspects such as student literacy, school infrastructure, and previous teaching experience, thus compromising implementation fidelity. Previous studies [60, 61] have also found that teachers tend to adapt curricula according to their students’ needs and vulnerabilities, such as reading proficiency and violence perpetration, which are prevalent issues in Brazilian public schools. In a study on teachers’ adaptations of kiR curricula, Miller-Day et al. [61] reinforced that, as much as such adaptations are expected, training must instruct teachers on how to adapt without changing the core aspects of each lesson.

According to the *Survey on the Use of Information and Communication Technologies in Brazilian Schools* [62], most public schools lack computer labs and technological infrastructure, such as laptops or tablets. As the PROERD curricula for fifth and seventh grade require audiovisual resources, school infrastructure is essential for program implementation as developed by kiR creators. Warren et al. [63] state that kiR videos are essential for interventions to achieve positive outcomes. As such, PROERD implementation fidelity cannot be expected to play an important role, since core elements such as video materials are not delivered as planned.

According to Medeiros et al. [64], teachers who deliver prevention programs feel overburdened by program activities, which require planning and classroom preparation which consequently affects their regular curricular activities. Thus, implementation of a school-based program by outside individuals is likely to be a positive experience for teachers and for the school itself. Although PROERD is delivered by police officers, the interviews show that these instructors are equally burdened by this role, as they continue to perform other policing functions. Given the importance of implementation quality [33], developers must focus on program viability to ensure that the required activities do not overburden instructors (from inside or outside the school setting) and compromise its quality [64].

In analyzing the interviews, it became evident that the null effect of implementation fidelity lies on the lack of cultural adaptation, as officers reported having to adapt the program to make implementation feasible due to student difficulties and school infrastructure.

### Limitations

A first limitation to consider is that the schools selected to participate in the RCTs were located in the low-income regions of São Paulo, which experience high exposure to drug use [65]; hence, instructors working in different areas could have distinct experiences and perceptions regarding the program. Since our sample consisted of schools that did not receive any intervention in the three years prior to the study to ensure non-contamination, the data cannot be generalized to all schools in São Paulo. Another limitation concerns the fidelity forms, which were subject to information bias due to self-reporting [29]. Moreover, both the questionnaire and fidelity forms were measured by dichotomous answers, limiting the analysis. One final limitation is that we didn’t use school achievement and other important predictors of drug use, such as mental health, family environment and drug access as control variables, which have strong predictive power for substance use.

## Conclusion

This study is the first to evaluate PROERD implementation fidelity in Brazil, highlighting important considerations to improve is effectiveness and sustainability. Future studies examining these parameters should employ better reliable instruments to measure implementation fidelity (dosage and quality), such as class observations. Moreover, future PROERD investigations must define which activity touches upon the core element of a lesson [61]. PROERD instructors with a clear understanding of the essential elements to be taught is key, allowing them to adapt less important parts in a structured manner according to the audience. Given the country’s territorial extent and its different realities, PROERD curricula must undergo a process of cultural adaptation. Moreover, the Military Police should incorporate the scientific findings regarding PROERD implementation in São Paulo and continue to investigate its effects not only in the city, but throughout the country.

## Electronic supplementary material

Below is the link to the electronic supplementary material.

Supplementary Material 1

## Data Availability

Data and materials are available upon request.

## Declarations

### Trial Registration

Study registered in the Brazilian Ministry of Health Register of Clinical Trials (REBEC) under protocol no. 6q23nk.

### Completeness

N = times when lesson activities were delivered. % = activities completed in each class.

### Alteration

N = times when lesson activities were altered. % = activities altered in each class.

## List of Abbreviations

ABEP: Brazilian Association of Research Companies.
cRCT: Cluster Randomized Controlled Trial.
DARE-kiR: DARE-Keepin’it REAL.
EU-DAP: European Drug Addiction Prevention Trial.
kiR: Keepin’ it REAL.
OECD: Organization for Economic Cooperation and Development.
PENSE: National Survey of School Health.
PROERD: Drug and Violence Resistance Educational Program (*Programa Educacional de Resistência às Drogas e à Violência)*.
RCT: Randomized Control Trial.
SES: Socioeconomic Status.
WHO: World Health Organization.
