# Supplementary material for: Implementation fidelity of a Brazilian drug use prevention program and its effect among adolescents: a mixed-methods study
Source: Subst Abuse Treat Prev Policy. 2022 Nov 1;17:71. doi: 10.1186/s13011-022-00496-w (PMC9623948; doi:10.1186/s13011-022-00496-w)

**Table S1**: Distribution of sociodemographic variables and patterns of drug use among 5th grade students participating in the PROERD cluster randomized controlled trial, according to the students included in the longitudinal analysis and those that were lost in the 9-month follow-up. Attrition analysis for covariates (N = 1,727).

| Sociodemographic variables and patterns of drug use | Losses** | | Follow-up* | | pª |
| --- | --- | --- | --- | --- | --- |
|  | (N = 393) | | (N = 1,334) | |  |
|  | N | % | N | % |  |
| Group |  |  |  |  |  |
| Control | 226 | 57.51 | 700 | 52.47 | 0.079 |
| Intervention | 167 | 42.49 | 634 | 47.53 |  |
| Sex |  |  |  |  |  |
| Female | 178 | 45.29 | 667 | 50.00 | 0.101 |
| Male | 215 | 54.71 | 667 | 50.00 |  |
| Age |  |  |  |  |  |
| 09 – 11 years | 352 | 89.57 | 1,301 | 97.53 | **<0.001** |
| 12 – 15 years | 41 | 10.43 | 33 | 2.47 |  |
| Baseline Drug Use |  |  |  |  |  |
| Alcohol |  |  |  |  |  |
| Lifetime drug use | 66/ 393 | 16.79 | 237/ 1,334 | 17.77 | 0.656 |
| Past-year drug use | 41/ 392 | 10.46 | 123 / 1,329 | 9.26 | 0.476 |
| *Binge drinking* |  |  |  |  |  |
| Lifetime drug use | 11 / 391 | 2.81 | 22/ 1,329 | 1.66 | 0.142 |
| Past-year drug use | 9 / 392 | 2.30 | 11 / 1,329 | 0.83 | **0.017** |
| Tobacco |  |  |  |  |  |
| Lifetime drug use | 9 / 389 | 2.31 | 22 / 1,333 | 1.65 | 0.387 |
| Past-year drug use | 3 / 389 | 0.77 | 9 / 1,333 | 0.68 | 0.841 |
| Marijuana |  |  |  |  |  |
| Lifetime drug use | 6 /388 | 1.55 | 4 / 1,333 | 0.30 | **0.004** |
| Past-year drug use | 2/ 388 | 0.52 | 2 / 1,333 | 0.15 | 0.188 |

* The cases answered the questionnaire at the beginning of the study and at 9 month follow-up.

** The cases answered the questionnaire only in the beginning.

a. Chi-Square Test.

**Table S2**: Distribution of sociodemographic variables and patterns of drug use among 7th grade students participating in the PROERD cluster randomized controlled trial, according to the students included in the longitudinal analysis and those that were lost in the 9-month follow-up. Attrition analysis for covariates ​​(N = 2,303).

| Sociodemographic variables and patterns of drug use | Losses** | | | Follow-up* | | pª |
| --- | --- | --- | --- | --- | --- | --- |
|  | (N = 564) | | | (N = 1.739) | |  |
|  | N | % | | N | % |  |
| Group |  |  | |  |  |  |
| Control | 267 | 47.34 | | 836 | 48.07 | 0.762 |
| Intervention | 297 | 52.66 | | 903 | 51.93 |  |
| Gender |  |  | |  |  |  |
| Female | 309 | 54.79 | | 878 | 50.49 | 0.076 |
| Male | 255 | 45.21 | | 861 | 49.51 |  |
| Age |  |  | |  |  |  |
| 10 – 13 years | 466 | 82.62 | | 1.660 | 95.46 | <0.001 |
| 14 – 17 years | 98 | 17.38 | | 79 | 4.54 |  |
| Baseline Drug Use |  | |  |  |  |  |
| Alcohol |  | |  |  |  |  |
| Lifetime drug use | 251/ 561 | | 44.74 | 615 / 1,737 | 35.41 | <0.001 |
| Past-year drug use | 138 / 560 | | 24.64 | 320 / 1,735 | 18.44 | 0.001 |
| *Binge drinking* |  | |  |  |  |  |
| Lifetime drug use | 85 / 560 | | 15.18 | 144 / 1,736 | 8.29 | <0.001 |
| Past-year drug use | 57 / 560 | | 10.18 | 75 / 1,736 | 4.32 | <0.001 |
| Tobacco |  | |  |  |  |  |
| Lifetime drug use | 44 / 560 | | 7.86 | 68 / 1,736 | 3.92 | <0.001 |
| Past-year drug use | 19 / 560 | | 3.39 | 17 / 1,735 | 0.98 | <0.001 |
| Inhalants |  | |  |  |  |  |
| Lifetime drug use | 71 / 559 | | 12.70 | 168 / 1,733 | 9.69 | 0.043 |
| Past-year drug use | 19 / 559 | | 3.40 | 38 / 1,733 | 2.19 | 0.111 |
| Marijuana |  | |  |  |  |  |
| Lifetime drug use | 45 / 560 | | 8.04 | 33 / 1,735 | 1.90 | <0.001 |
| Past-year drug use | 24 / 560 | | 4.29 | 17 / 1,735 | 0.98 | <0.001 |
| Cocaine |  | |  |  |  |  |
| Lifetime drug use | 2 / 559 | | 0.36 | 7/ 1,733 | 0.40 | 0.879 |
| Past-year drug use | 1/ 559 | | 0.18 | 1/ 1,733 | 0.06 | 0.399 |

* The cases answered the questionnaire at the beginning of the study and at 9 month follow-up.

** The cases answered the questionnaire only in the beginning.

a. Chi-Square Test.

**Table S3:** Characteristics of interviwees (n = 19).

| Interviewees | Sex (F/M) | Age | Education level^b^ | Position in police hierarchy^a^ | Years teaching PROERD |
| --- | --- | --- | --- | --- | --- |
| P1 | F | 42 | - | Cabo | 5 |
| P2 | F | 26 | High School | Soldado | 1 |
| P3 | M | 32 | High School | Cabo | 5 |
| P4 | M | 45 | High School | Cabo | 14 |
| P5 | M | 40 | High School | Cabo | 15 |
| P6 | M | 38 | High School | Cabo | 11 |
| P7 | M | 42 | Universitary | Cabo | 6 |
| P8 | M | 32 | High School | Soldado | <1 |
| P9 | M | 42 | High School | Cabo | 12 |
| P10 | M | 39 | High School | Cabo | 4 |
| P11 | F | 47 | High School | Cabo | 10 |
| P12 | F | 40 | High School | Cabo | 2 |
| P13 | M | 34 | High School | Soldado | 4,5 |
| P14 | M | 33 | Under graduation | Cabo | 1 |
| P15 | M | 46 | High School | Cabo | 9 |
| P16 | M | - | High School | Cabo | 9 |
| P17 | F | 48 | Under graduation | Cabo | 19 |
| P18 | F | 48 | Under graduation | Cabo | 15 |
| P19 | F | 49 | Graduation | Cabo | 10 |

a-Soldado is the lowest level in the Military Police hierarchy. Cabo is the following level. So, Soldado and Cabo are the two lowest level in the hierarchy.

b- We have transfomed the Brazilian category to the US pattern. So High School includes also the officers that have started a college but did not received a degree. Undergradution is the first degree that universities offer and Graduation is the higher level (master and doctoral degrees).

**Anex S1:** Semi-structured interview script for police officers

Sex, age, education level, police rank, years teaching PROERD.

**Initial Context**

1. How and why did you join PROERD?

In your opinion, what are the effects of PROERD on participating children (5th and 7^th^ grades) and how do you perceive it?

**Relations**

1. What do you think about the alignment between school management and classroom work for program implementation? Conveniences and Difficulties.
2. How can these relationships influence the program effects?

**Program Implementation**

1. Was the received training sufficient? Why?
2. How does it (the training) influence your practice? Why?
3. What is your opinion about the manuals? Do you think they is suitable for the age groups?
4. How does the manual support class planning?
5. Is the program content suitable for the age groups?
6. What does and does not work for the students? What are the conveniences and difficulties found in the classroom? What activities are most accepted and least accepted by students?
7. Is it different to implement the curricula for the fifth and seventh grades? Why?
8. How does the demands of the program implementation (class planning, relationship with the principals and pedagogical coordination) influence your daily life as a police officer and vice vesa (how does your daily life as a police officer influence the program implementation)?

**Sugestions**

1. What would you change in the program?

What are the lessons learned that can help expanding the project?

**Figure S1**. Code tree including the used and non-used codes for the qualitative analysis.


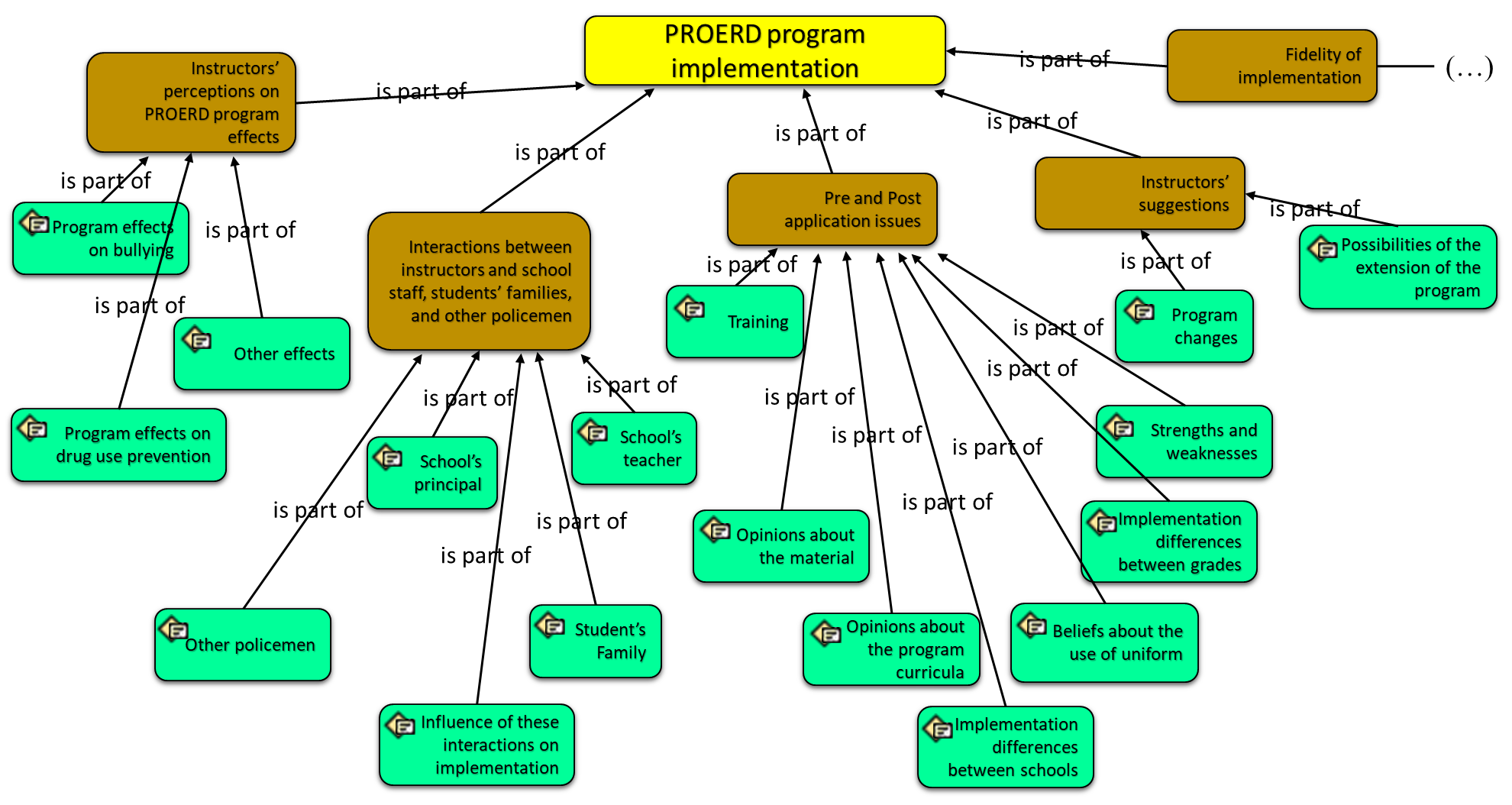


**Figure S1.** Continue…


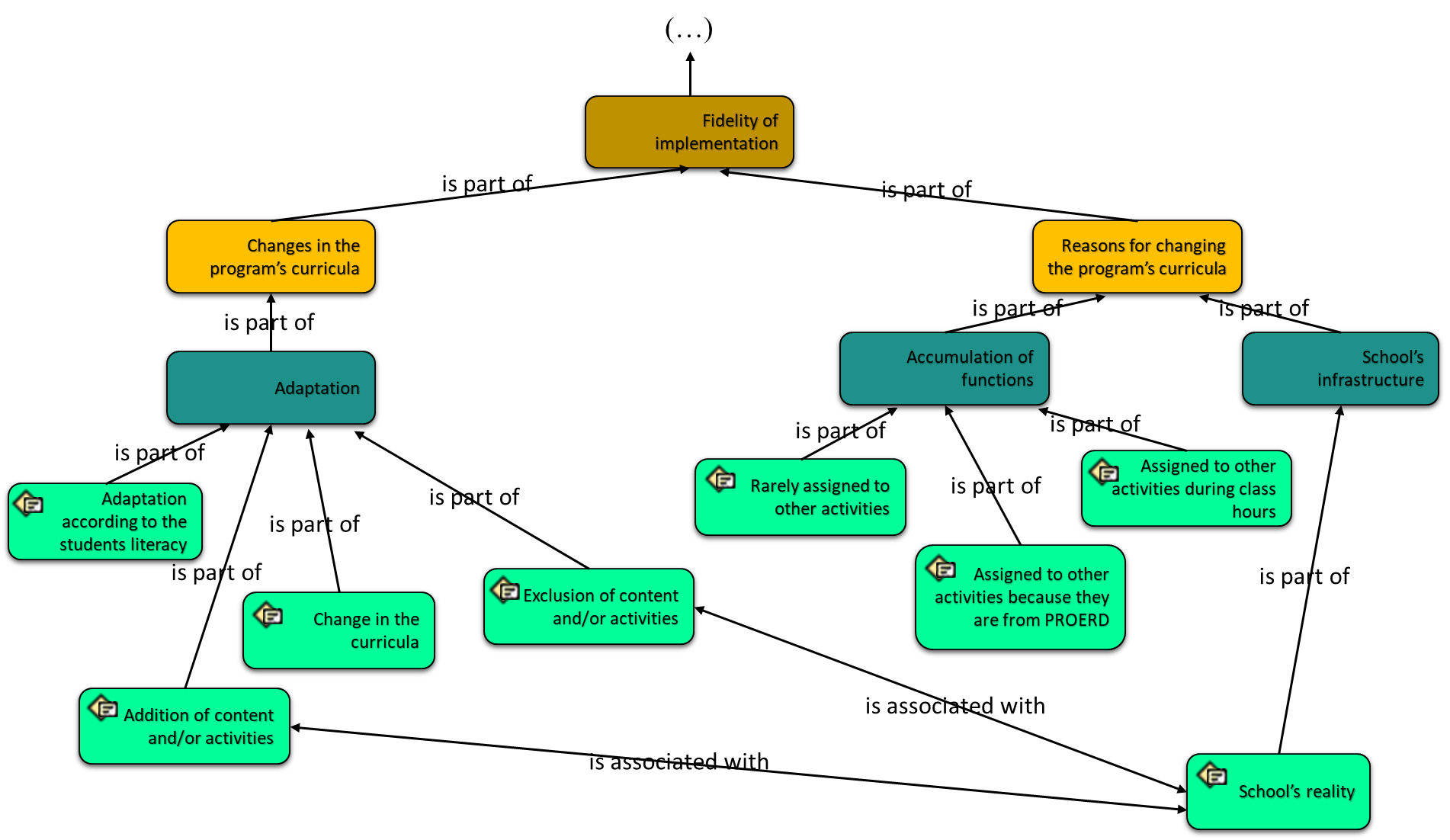

Supplement: Supplementary file 1 — Supplementary Material 1 [file 13011_2022_496_MOESM1_ESM.docx]
